# Nosocomial cluster of carbapenemase-producing *Enterobacter cloacae* in an intensive care unit dedicated COVID-19

**DOI:** 10.1186/s13756-021-01022-6

**Published:** 2021-10-21

**Authors:** Guillaume Miltgen, Thomas Garrigos, Pascal Cholley, Marine Deleume, Nicolas Allou, Jérôme Allyn, David A. Wilkinson, Nathalie Lugagne, Olivier Belmonte, Xavier Bertrand, Didier Hocquet, Patrick Mavingui

**Affiliations:** 1 Laboratoire de Bactériologie, CHU Félix Guyon, Allée des Topazes, 97400 Saint-Denis, La Réunion France; 2 UMR PIMIT, Processus Infectieux en Milieu Insulaire Tropical, CNRS 9192, INSERM U1187, IRD 249, Université de La Réunion, Sainte-Clotilde, La Réunion France; 3 Service d’Hygiène Hospitalière, CHU Jean Minjoz, Besançon, France; 4 UMR CNRS 6249 Chrono-Environnement, Université de Bourgogne Franche-Comté, Besançon, France; 5 Service de Réanimation Polyvalente, CHU Félix Guyon, Saint-Denis, La Réunion France; 6 Service d’hygiène hospitalière, CHU Félix Guyon, Saint-Denis, La Réunion France

**Keywords:** *Enterobacter cloacae*, Carbapenemase, Cross transmission, Personal protective equipment, Intensive care unit, COVID-19

## Abstract

Concomitant prevention of SARS-CoV-2 and extensively drug-resistant bacteria transmission is a difficult challenge in intensive care units dedicated to COVID-19 patients. We report a nosocomial cluster of four patients carrying NDM-1 plasmid-encoded carbapenemase-producing *Enterobacter cloacae*. Two main factors may have contributed to cross-transmission: misuse of gloves and absence of change of personal protective equipment, in the context of COVID-19-associated shortage. This work highlights the importance of maintaining infection control measures to prevent CPE cross-transmission despite the difficult context and that this type of outbreak can potentially involve several species of *Enterobacterales.*

**Supplementary Information:**

The online version contains supplementary material available at 10.1186/s13756-021-01022-6.

## Introduction

The SARS-CoV-2 pandemic and antimicrobial resistance are two major public health problems that have so far rarely been intertwined [1, 2]. Unfortunately, in regions with high prevalence of carbapenemase-producing *Enterobacterales* (CPE) such as the Southwest Indian Ocean area (SIOA), some tertiary hospitals may be simultaneously confronted with both phenomena [3]. We describe here a nosocomial cluster of NDM-1 carbapenemase-producing *Enterobacter cloacae* (NDM-1 *Ec*) in an intensive care unit (ICU) of the University Hospital of Reunion Island (UHRI).

Reunion Island is a French overseas territory located close to Madagascar with similar healthcare standards to those found in mainland France. Patients with COVID-19 hypoxemic pneumonia from the SIOA requiring ICU are evacuated to the UHRI, considered as the reference hospital in the region. During the SARS-CoV-2 epidemic, one of the three subunits of the ICU (subunit C) was dedicated to suspected or confirmed COVID-19 patients.

## Methods

### Infection control policies and CPE screening

The ICU of the UHRI consists of three subunits (A, B and C), each containing 10 single rooms. UHRI's policies consist in accordance to the French guidelines of isolation precautions and screening of all admitted patients coming from a foreign country (including Mayotte Island) or recently hospitalized in another healthcare facility. In addition, patients hospitalized in the ICU were systematically screened for carriage of multidrug-resistant microorganisms, upon admission and weekly thereafter. Patients were kept on contact precautions until the first negative result was obtained. Furthermore, excreta management was optimally handled with bedpans equipped with gel bags and additionally with protective bags in case of CPE colonization (“Care Bag” type); and washed between each use in a bedpan washer. Regular meetings and audits were organized between the intensive care and infection control teams to evaluate these infection prevention practices. CPE screening was performed on rectal swabs by culture on selective chromogenic agar (ChromID CARBA Smart, BioMérieux, Marcy l’Etoile, France) according to the manufacturer’s recommendations.

### CPE identification and antimicrobial susceptibility testing

Bacterial species was identified using MALDI-TOF mass spectrometry (Microflex, Bruker daltonics, Bremen, Germany). Carbapenemase production was confirmed using immuno-chromatographic assay (NG Rapid Test CARBA-5, NG Biotech, Guipry, France) and multiplex PCR (Xpert Carba, Cepheid, Sunnywale, USA). Antibiotic susceptibility testing was performed by agar diffusion according to the 2020 EUCAST recommendations (www.eucast.org).

### Molecular genotyping and bioinformatics analysis

Clonality between isolates was evaluated by pulsed-field gel electrophoresis and multi-locus sequence typing as previously described [3]. Conjugation experiments were performed using the azide-resistant laboratory strain *Escherichia coli* J53 as previously described [4]. Plasmid DNA of the transconjugant was extracted using the QIAGEN Maxiprep kit according to the manufacturer’s recommendations. Sequencing of the extracted plasmid and of the genome of *E. cloacae* donor strain was performed on the MinION using an R9 (FLO-MIN106) flowcell. Antibiotic resistance genes were detected by querying the ResFinder database v. 4.0 [5]. The plasmid sequence generated is available on the Genbank database under the accession number MW464182. The detailed bioinformatics protocol is supplied in the supplementary material (Additional file 1).

## Results

**Fig. 1:**
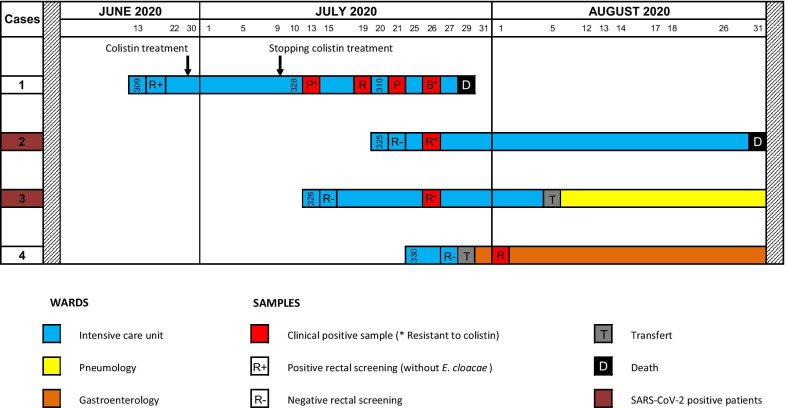
Timeline of patients infected/colonized with NDM-1 producing *Enterobacter cloacae* (NDM-1 *Ec*) hospitalized in intensive care unit of the University Hospital of Reunion Island, Saint-Denis, France, June–August 2020 (*n* = 4). Each NDM-1 *Ec* positive patient is represented by a line and each positive sample is symbolized by a red box. For the positive clinical samples, letters in the red boxes indicate the specimen source: B for blood origin, P for pulmonary origin and R for rectal origin. Numbers in the boxes are those of the room in ICU. The death of Case 2 was considered not attributable to NDM-1 *Ec.*

Case 1, considered as the index case, was hospitalized in ICU on 2020 June 13 (COVID-19 suspected pneumonia). He was positive for *Escherichia coli* and *Klebsiella pneumoniae* both producing NDM-1 on admission and for NDM-1 *Ec* for the first time on July 13, one month after its admission (Fig. 1). During July and August 2020, three other patients hospitalized in adjacent rooms tested positive for NDM-1 *Ec*. Case 1, originating from Reunion Island, died from bacteremia with NDM-1 *Ec*; the other three cases were colonized. Cases 2 and 3 had been transferred from Madagascar and were hospitalized for acute respiratory distress due to COVID-19. Case 4 came from Reunion Island and was hospitalized for 3 days in subunit C for a digestive haemorrhage (due to saturation of the 2 other subunits). The systematic screening on admission was negative for Cases 2, 3 and 4. During the period of the outbreak, 201 patients were hospitalized in the ICU, of which 94 were hospitalized in subunit C; all (exept the four cases) were tested negative for NDM-1 *Ec*. A total of seven isolates were collected (Fig. 1). Three isolates were not susceptible to all β-lactams (including cefiderocol), fluoroquinolones, sulfamethoxazole-trimethoprim, amikacin, and four were additionally resistant to colistin (Minimal Inhibitory Concentration—MIC, 16 mg/L) and to tigecycline (MIC, 2 mg/L). They remained susceptible only to gentamicin (MIC, 1 mg/L), fosfomycin (MIC, 16 mg/L) and the combination of aztreonam and avibactam (cumulated MIC, 0.5 mg/L).

**Fig. 2:**
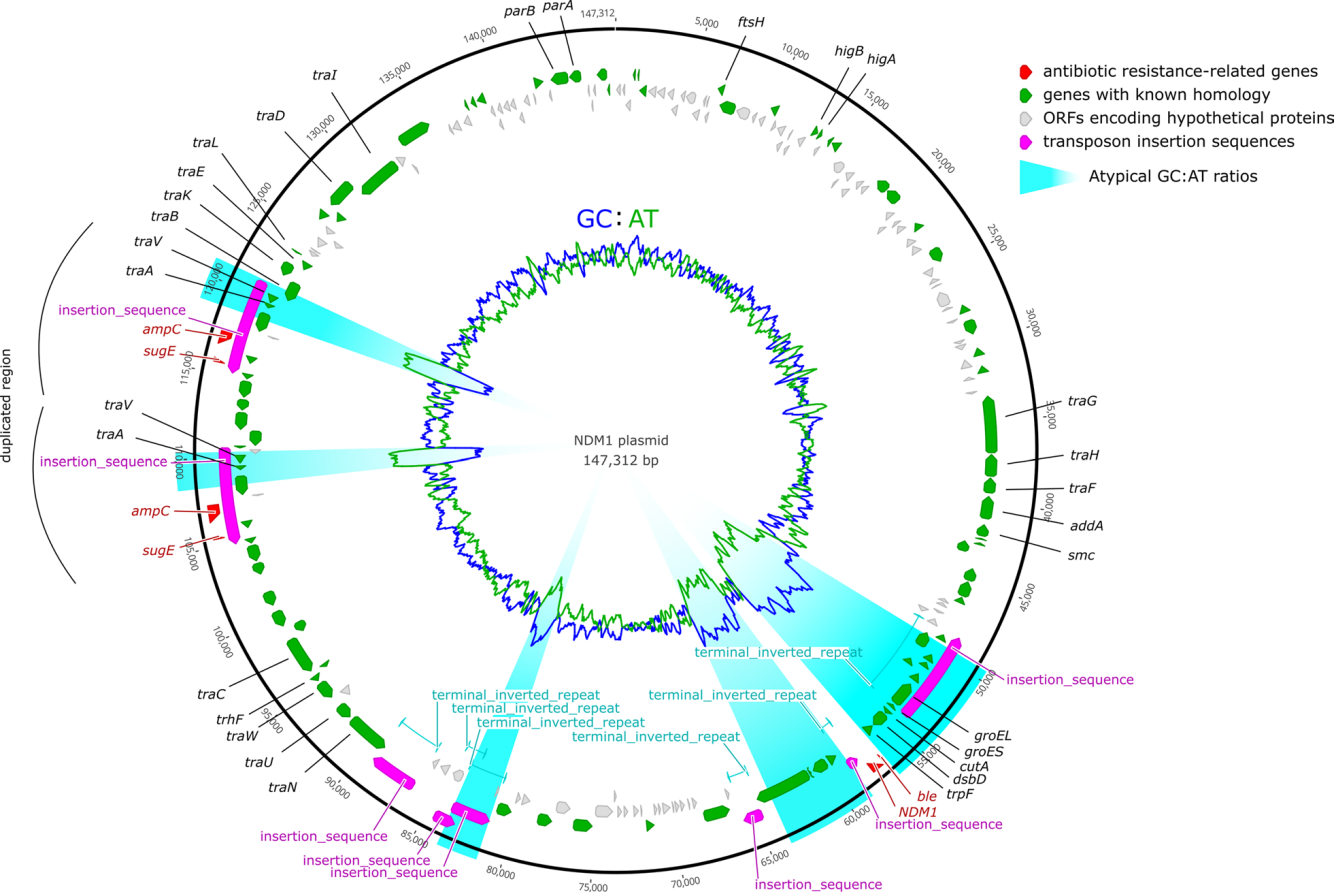
Schematic map of the putative NDM-1 containing plasmid identified by conjugative transfer and MinION long-read sequencing. Genes associated with plasmid replication and maintenance as well as mobile elements related to the acquisition of antibiotic resistance are labelled. Other gene labels are left off for clarity. The plasmid carried 2 insertion sequences included resistances gene: (1) a truncated IS*Aba*125 with *bla*_NDM-1_ and *ble* genes and (2) a duplicated region with *ampC* and *sugE* genes, interspersed between components of the *tra* operon

The analysis of five isolates of NDM-1 *Ec* (two isolates—one susceptible and one resistant to colistin—were analyzed from Case 1) shared the same pulsotype and belonged to Sequence Type 90. Resistance to carbapenem could be transferred by conjugation to the *E. coli* J53. The resistance gene *bla*_NDM-1_ was carried by an IncC plasmid of 147,312 bp and associated with the bleomycin resistance gene *ble* in a truncated IS*Aba125* insertion sequence (Fig. 2). This plasmid also harbored a duplicated region with *ampC* and *sugE* genes (3rd cepholosporin and quaternary ammonium resistances, respectively), interspersed between components of the *tra* operon (Fig. 2). The resistance mechanism to colistin, especially mutations in the PmrA/B and PhoP/Q two-component systems, is being explored.

## Discussion

A cluster of CPEs shared by four patients is unusual in this ward, where paramedical staff are well trained to preventive measures of cross-transmission and where enhanced contact precautions often need to be applied for medical evacuation [6]. Adherence of the ICU’s health care workers to these preventive practices had been regularly and favourably evaluated by the infection control team. In our opinion, two main factors could have favoured the transmission of NDM-1 *Ec*—most likely imported into the ICU by the Case 1—to the other three patients: (1) the misuse of gloves in nursing acts, and (2) the absence of change of over-gowns between patient rooms, considering that the whole subunit was as a COVID-positive sector. This way of using personal protective equipment (PPE) was implemented to limit the risk of contamination of nursing staff during undressing and to reduce demand in a context of a PPE shortage. Concomitant positive screening of Cases 2, 3, and 4 within a short time interval of seven days corroborates the probable health-care workers carrying transmission (Fig. 1). As the ratio of healthcare workers to patients remained constant during this period, the overload of the teams probably did not favor this cross-transmission. Moreover, these three patients had a negative rectal screening at their admission into the ICU, confirming the nosocomial transmission.

We immediately implemented infection control measures including (1) simultaneous screening of all patients in the subunit and (2) strict application of enhanced contact precautions with glove and over-gown change between each room. In addition, a hygienist nurse was assigned to the ICU to advise and retrain the nursing staff in the preventive measures of cross-transmission. Since, no new cases of infection or colonization with this NDM-1 *Ec* clone have occurred to date, three months after the episode.

NDM carbapenemase-producing *E. cloacae* ST90 clone has been very rarely described in the literature. Hence, only one study refers to an NDM-1 carbapenemase-producing *E. cloacae* ST90 from Henan Province in China [7]. This ST has also been described as potential vector of multidrug resistance by being associated with the *bla*_VIM-2_ and *bla*_VIM-4_ carbepenemase-encoding genes in Argentina and Poland, respectively [8, 9]. Although the carbapenemase-encoding gene may be carried by the same species of *Enterobacterales* (as described here), one should be aware that carbapenemase-resistance determinants are highly transferable *via* mobile genetic elements and can easily spread to other co-occurring species of the gut flora, like *K. pneumoniae* or *E. coli*, as in Case 1, and described elsewhere [10]. IncC plasmids (as well as IncFII and IncX3) are well known to be the genetic carrier and a vector for transmission of *bla*_NDM_ genes between *Enterobacterales* [11–13]. As recently stated in an update of the French national guidelines, it is therefore important to consider the type of enzyme produced by the enterobacteria (such as OXA-48-like or NDM) independently of the bacterial species to define whether patients are part of the same outbreak [6]. Lastly, the adaptive resistance to colistin of the NDM-1 *Ec* isolate found in Case 1 was probably induced, as this patient had been treated with colistin (Fig. 1).

## Conclusions

Here, we point out a new situation where the admission of COVID-19 patients can influence the long-established infection control behaviours. Such “collateral damage” has recently been described by authors who investigated the first nosocomial clusters in COVID-19 units [14–16]. Our observation highlights the importance of maintaining measures to control CPE despite the additional pressure put on ICUs by the large number of incoming COVID-19 cases. Finally, clinicians and hygienists should be aware that an CPE’s outbreak can affect several species of *Enterobacterales* due to the high transferability of the carbapenemase-encoding resistance genes.

## Supplementary Information

**Additional file 1**. Detailed bioinformatics analyses.

## Data Availability

All data generated or analysed during this study are included in this published article and its supplementary information files.
