# Supplementary material for: Nosocomial cluster of carbapenemase-producing Enterobacter cloacae in an intensive care unit dedicated COVID-19
Source: Antimicrob Resist Infect Control. 2021 Oct 21;10:151. doi: 10.1186/s13756-021-01022-6 (PMC8529563; doi:10.1186/s13756-021-01022-6)
Supplement: Supplementary file 1 — Additional file 1. Detailed bioinformatics analyses. [file 13756_2021_1022_MOESM1_ESM.docx]

**Detailed bioinformatics analyses**

Extracted plasmid and total genomic DNA were quantified using the Qubit™ dsDNA HS Assay kit, and sequencing libraries were prepared from 400 ng of genetic material using the Oxford Nanopore Technologies Rapid Barcoding Kit (SQK-RBK004). Barcodes RB01-RB03 were used for biological samples, barcode RB04 was used with a water control. Sequencing was performed on the MinION using an R9 (FLO-MIN106) flowcell, overnight for a total of 10 hours. Raw data were basecalled using the guppy basecaller (version 4.0.11) with quality filtering in high accuracy mode and then demultiplexed and barcodes trimmed using the guppy barcoder (version 4.0.11). Insignificant numbers of reads were obtained from the negative control. Sequence data were assembled using the CANU *de novo* assembler (version 1.9) [1], which also predicted likely circularity of assembled contigs. Circular construct breakpoints were determined by self-mapping using NUCleotide MUMmer (version 3.1) [2]. Antibiotic resistance genes were detected by querying the ResFinder database [3]. Contigs were annotated using a combination of pairwise comparison to previously annotated NDM-containing plasmids in the NCBI database using Geneious (version 9.1.8) and prokka (version 1.14.6) [4] followed by annotation verification against the eggnog-mapper database [5]. Transposon insertion sites were predicted using the ISEScan algorithm [6], and GC ratios were examined in Geneious (version 9.1.8).

The plasmid sequence generated as part of this study is available on the Genbank database under the accession number MW464182.

1. Koren S, Walenz BP, Berlin K, Miller JR, Bergman NH, Phillippy AM. Canu: Scalable and accurate long-read assembly via adaptive κ-mer weighting and repeat separation. Genome Res. 2017;27: 722–736.

2. Kurtz S, Phillippy A, Delcher AL, Smoot M, Shumway M, Antonescu C, *et al.* Versatile and open software for comparing large genomes. Genome Biol. 2004;5: R12.

3. Bortolaia V, Kaas RS, Ruppe E, Roberts MC, Schwarz S, Cattoir V, *et al.* ResFinder 4.0 for predictions of phenotypes from genotypes. J Antimicrob Chemother. 2020.

4. Seemann T. Prokka: rapid prokaryotic genome annotation. Bioinformatics. 2014 Jul 15;30(14):2068-9.

5. Huerta-Cepas J, Forslund K, Coelho LP, Szklarczyk D, Jensen LJ, Von Mering C, *et al.* Fast genome-wide functional annotation through orthology assignment by eggNOG-mapper. Mol Biol Evol. 2017 Aug 1;34(8):2115-2122.

6. Xie Z, Tang H. ISEScan: automated identification of insertion sequence elements in prokaryotic genomes. Bioinformatics. 2017;33: 3340–3347.
